# Postoperative Long-term Outcomes of Patient with Craniopharyngioma Based on CyberKnife Treatment

**DOI:** 10.7759/cureus.7207

**Published:** 2020-03-08

**Authors:** Genichiro Ohhashi, Shinichiro Miyazaki, Hidetoshi Ikeda, Tomokatu Hori

**Affiliations:** 1 Neurosurgery, Koyu Neurosurgery and Ophthalmology Hospital, Sagamihara, JPN; 2 CyberKnife Center, Shinyurigaoka General Hospital, Kawasaki, JPN; 3 Pituitary Diseases, Research Institute for Pituitary Disease, Southern Tohoku General Hospital, Koriyama, JPN; 4 Neurosurgery, Moriyama Neurological Center Hospital, Tokyo, JPN

**Keywords:** craniopharyngioma, stereotactic radiotherapy, long-term outcome, conservative surgery, cyberknife

## Abstract

Objective

The results of CyberKnife treatment in patients with craniopharyngiomas are excellent, but reports of long-term follow-up are rare. Hence, considering the possibility of a long-term follow-up of five years or more, we examined the long-term prognoses of these patients.

Materials and Methods

Of 33 patients, 12 were males and 21 were females. On postoperative evaluation, three patients experienced recurrence after total resection and were treated using CyberKnife. Twenty-five patients were treated with CyberKnife after partial resection. The mean age at treatment was 47 years, and the follow-up period was 61 to 129 months.

Results

Of the cases assessed as totally resected in the postoperative evaluation, three recurred after 18 months. CyberKnife treatment was administered immediately in recurrent cases; subsequently, no recurrences were observed for 25 months or more. No recurrences were observed in any patients treated with CyberKnife on the residual site after surgical treatment. Many cases had improved pituitary function, but none had deteriorated. In addition, no case of visual function deterioration was reported.

Conclusion

Twenty years have passed since the introduction of CyberKnife treatment; however, only a few reports have examined the long-term prognosis of patients with craniopharyngiomas who underwent this treatment. We have been aware of the efficacy of CyberKnife treatment for ten years or more; its long-term results are evident, and the good growth control and low adverse effects are impressive. We are confident that we can maintain good treatment results by combining conservative surgical resection with minimal complications and CyberKnife treatment for new patients in the future.

## Introduction

Craniopharyngioma is a tumor that originates from the diencephalic and pituitary regions. As the genetic abnormalities responsible for craniopharyngioma have been revealed, craniopharyngioma has been identified as an acquired tumor based on somatic mutations (β-catenin and BRAF) in recent years [1-2]. This tumor is histologically benign, and its overall annual incidence is 1.3 per one million people. According to the National Cancer Database, the five-year survival rate is 80% and decreases with increasing age at diagnosis. The survival rate is high in children and reported to have been improving in recent years [3].

In adults, the characteristic initial symptoms include local symptoms such as visual impairment, diabetes insipidus, and neurological dysfunction, as well as symptoms associated with hypopituitarism initially presenting as adult growth hormone deficiency. Although surgical resection is the first choice of treatment, ensuring total resection without causing complications is difficult because the optic nerves and important blood vessels such as the internal carotid, anterior communicating, and anterior cerebral arteries exist around the tumor and also because resection of the upper part of the tumor is associated with the risk of damage to the hypothalamus. Therefore, long-term multidisciplinary follow-up is necessary for all the patients [4-5].

Consequently, a combination of partial resection and radiotherapy is considered the second choice. The addition of radiotherapy contributes to increased tumor control and decreased mortality [6-8]. When fractionated irradiation is performed after partial resection, the ten-year survival rate is as high as 90% or more. However, the control rate with conventional radiotherapy is not sufficient. In addition, radiotherapy has been associated with adverse events such as hypopituitarism and visual impairment [9]. CyberKnife treatment, a type of stereotactic radiotherapy, is an established therapy that allows selective irradiation of only tumors and the prevention of damage to surrounding important organs. Although the efficacy of CyberKnife treatment for craniopharyngioma has been reported, reports on long-term prognosis only include a small number of cases [6]. Moreover, few sufficient follow-up studies have reported on the long-term control rate and adverse reactions to radiotherapy [10].

This follow-up study was conducted in patients who received combination therapy with surgery and CyberKnife and could be followed up for ≥5 years. The main objective was to establish the therapeutic requirements and effects and long-term outcomes of CyberKnife treatment for craniopharyngioma.

## Materials and methods

The authors encountered 33 patients with craniopharyngioma during a five-year period from 2009 to 2014 at the Shinyurigaoka General Hospital; recurrent cases and children were excluded. The patients consisted of 12 men and 21 women whose ages ranged from 29 to 77 years (median: 47.2 years). Among the patients, five were treated with surgery alone and the other 28 were treated with a combination of surgery and CyberKnife treatment.

In this study, the long-term outcomes in these 33 patients were evaluated. Patients were informed of this study in writing and all were approved. The initial symptoms were headache in 20 patients, hypopituitarism in 25, and visual dysfunction in 18. None of the patients were asymptomatic. Among the patients with hypopituitarism, 17 (61%) were diagnosed with severe adult growth hormone deficiency as defined by a growth hormone level of ≤9 ng/mL, measured with the endocrine diagnostic technique using growth hormone-releasing peptide-2. In addition, eight patients had decreased sex hormone levels, eight had hypothyroidism, three had diabetes insipidus that required treatment, and ten required adrenocortical hormone replacement therapy since before surgery.

The visual function test was performed by neuro-ophthalmologists in all the patients and revealed that nine patients had bitemporal hemianopsia, six had homonymous hemianopia, and three had patchy visual field defects. The severity of pain in patients with a chief complaint of headache was evaluated using the sixth version of the headache impact test (HIT-6). The scores ranged from 57 to 68 (mean, 60.3), indicating pain levels that interfered with daily living. The tumor volume targeted for CyberKnife treatment ranged from 1.75 to 14.72 cc (median, 6.2 cc), as determined by CyberPlan during treatment planning.

The radiosurgery system manufactured by Accuray Incorporated (Sunnyvale, CA) was used to administer the CyberKnife treatment with hypofractionation of three to eight fractions (mean, 5.8 fractions) at a peripheral dose of 2000 to 2550 cGy (2280 cGy/isodose% [D95], 76.9%). The mean prescribed dose for a single fraction of radiation was 1366 cGy. The volume of treated tumors ranged from 1.75 to 14.72 cc (mean, 6.2 cc). A conformal plan was developed for radiotherapy in all the patients. Tumors were located in the intrasellar region in three patients, the suprasellar region in eight, and both sides of the sella turcica in 22. The pathological diagnoses were squamous papillary craniopharyngioma in 30 patients and adamantinomatous craniopharyngioma in three. The follow-up period from initial treatment ranged from 61 to 109 months (mean, 80.0 months). During the follow-up period, one patient died from adrenal insufficiency associated with severe pneumonia at 73 months after the initial treatment.

## Results

**Figure 1:**
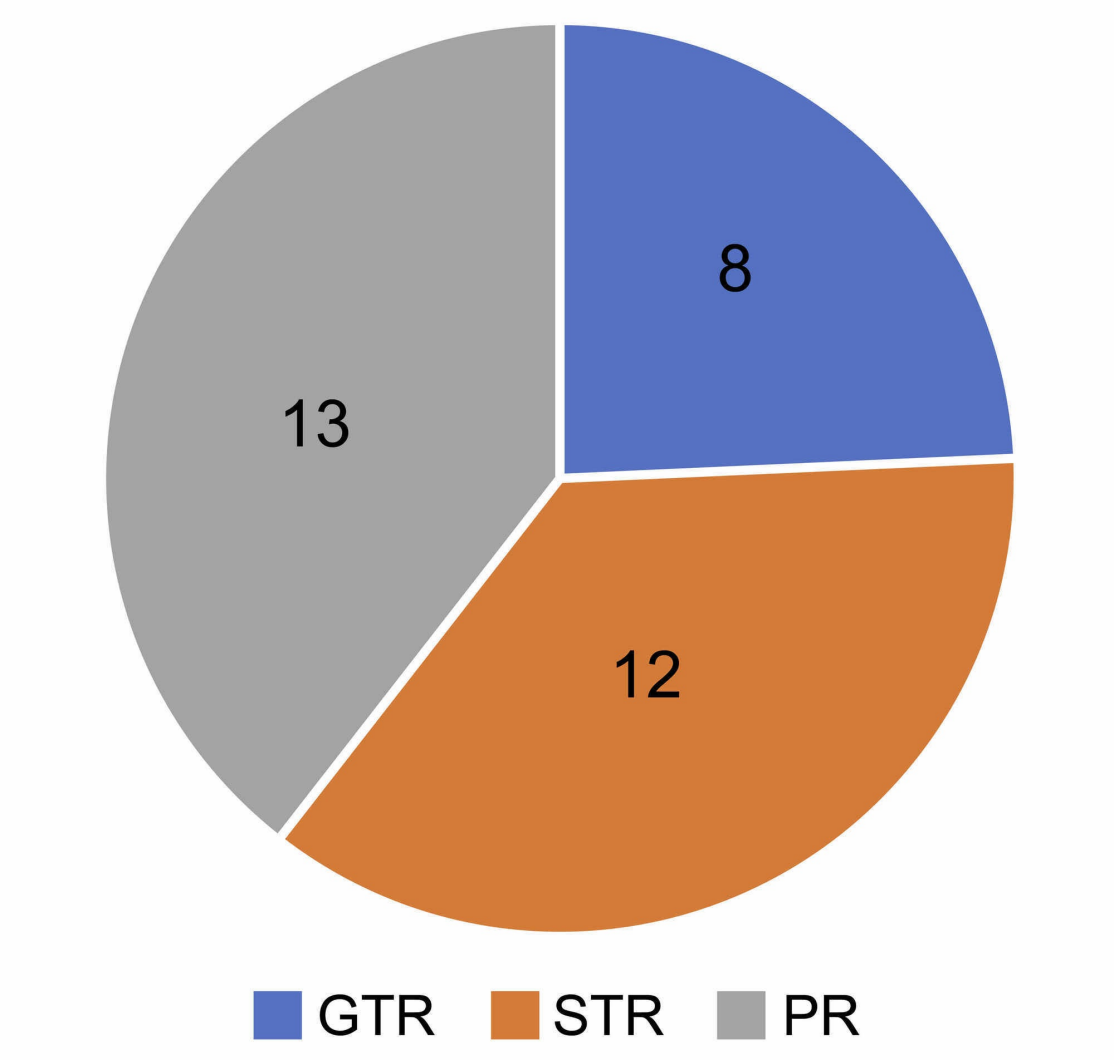
Surgical outcomes extended endonasal transsphenoidal approach Gross total removal (GTR) is 8 cases, subtotal removal (STR) is 12 cases, and partial removal (PR) is 13 cases.

Surgery was the first choice of treatment for 33 patients. In all the patients, surgery was performed via the extended endonasal transsphenoidal approach using the combined microscopic-endoscopic technique under a navigation system. Gross total resection was achieved in eight patients and subtotal or partial resection in 25. CyberKnife treatment was administered to residual tumor tissues within three months after surgery in 25 patients after excluding those determined to have undergone total resection. In addition, among the patients determined to have undergone total resection, three were confirmed to have recurrence using magnetic resonance imaging (MRI) during follow-up, and CyberKnife treatment was immediately performed. The outcomes of surgery performed using the extended transsphenoidal approach were total resection in eight patients, subtotal resection in 12, and partial resection in 13 (Figure 1).

In surgery, resection was terminated when abrasion was difficult to perform under microscopic or endoscopic visualization, or when vascular injury or hypothalamic disorder was predicted because abrasion would be blindly performed even under endoscopic visualization. In addition, the pituitary gland and hypophysial stalk were preserved in all the patients. No perioperative complications were observed, except diabetes insipidus, which was transient or lasted for up to approximately six months. Furthermore, besides the patients who had hypopituitarism before surgery, none of the patients required new hormone replacement therapy. Visual dysfunction was improved after surgery in all the patients, although the degree of improvement varied. For postoperative follow-up, 3-T MRI was performed once every three to six months. Of the eight patients who were determined to have undergone total resection, three were found to have enhanced cystic lesions at 18, 24, or 27 months. These lesions were determined to be recurrences, and CyberKnife treatment was immediately performed. In these three patients, no further recurrence was observed during the follow-up of 24 months or more. One patient who had been treated with CyberKnife after partial resection died from a combination of pneumonia and adrenal insufficiency six years after the treatment. In the remaining two patients, the visual field defect was aggravated by cyst enlargement soon after CyberKnife treatment, and the cysts were surgically opened. After cyst fenestration, the visual field defect immediately improved.

**Figure 2:**
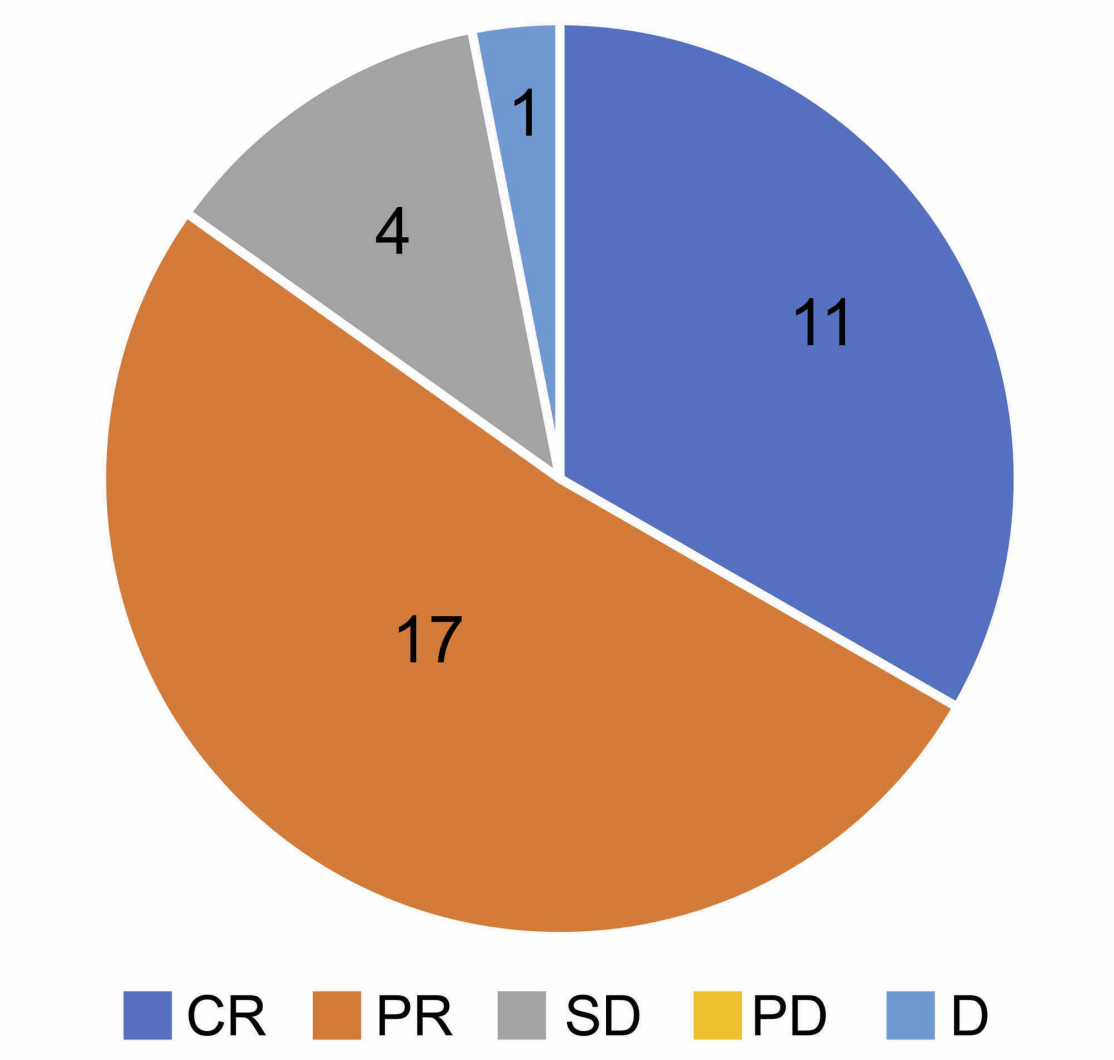
Overall treatment outcomes of combined surgery and CyberKnife therapy is shown based on revised RECIST guideline (version 1.1) There were 11 cases of complete response, 17 cases of partial response, four cases of stable disease, no progressive disease, and one case of death.

According to the assessment based on the guidelines proposed by New Response Evaluation Criteria in Solid Tumours: Revised RECIST Guideline (version 1.1), the outcomes in the 33 patients included in this study were complete remission in 11 patients, partial remission in 17, stable disease in four, progressive disease in zero, and death in one (Figure 2) [11]. 

**Figure 3:**
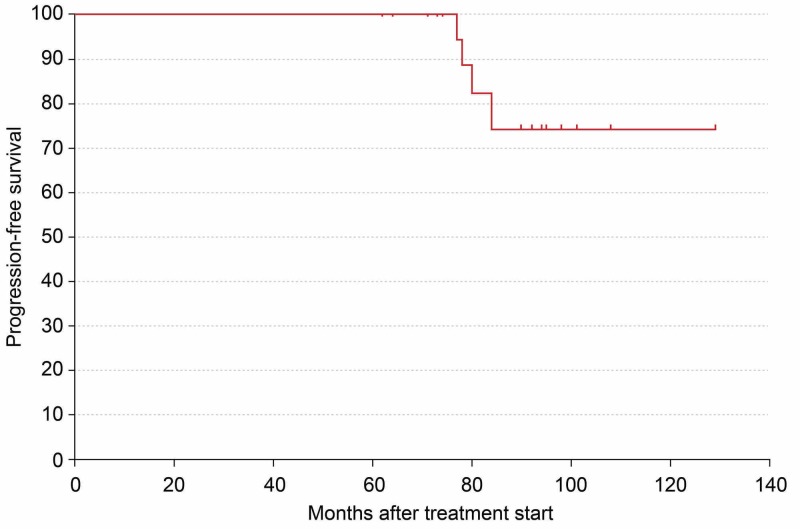
Analysis of the results of therapy using the Kaplan–Meier method showing progression-free survival rate as 74.8%

**Figure 4:**
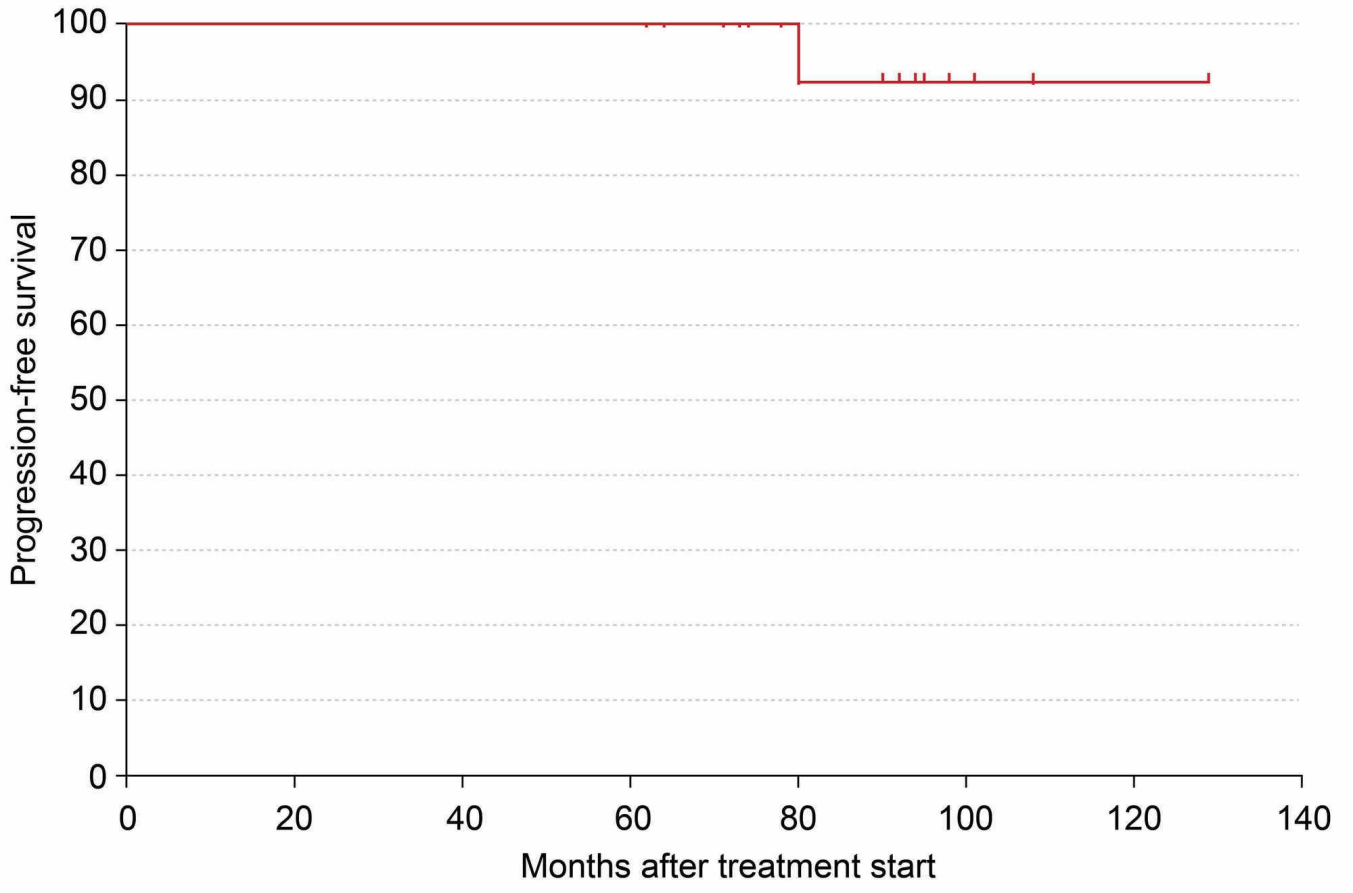
Analysis of therapy results using the Kaplan–Meier method showing local control rate as 94.1%

When these results were analyzed using the Kaplan-Meier method, the progression-free survival rate was 74.8% (Figure 3), and the local control rate was 94.1% (Figure 4).

## Discussion

The surgical procedure selected for craniopharyngioma has shifted from craniotomy, which had been widely performed, to the extended transsphenoidal approach. The latter has contributed to the reduction in the risk of complications [12]. However, no significant difference in total resection rate was found between the two procedures [13]. The previously reported incidence of adverse events associated with surgery ranged from 4% to 30% for deterioration of visual function, from 52% to 66% for deterioration of anterior pituitary function, from 25% to 45% for new onset of diabetes insipidus, from 7% to 10% for cerebrospinal fluid leak, and from 18% to 33% for memory disturbance [8,13-15]. In addition, as for the postoperative activities of daily living, the proportion of patients with a Karnofsky Performance Status of ≥80% was 83.8%, and the proportion of patients who attended school or work was 73.0%. Although craniopharyngioma is a benign tumor, surgical outcomes are not satisfactory. The rate of preservation of the hypophysial stalk, which depends on the policies of operators, widely varies from 41.5% to 97%, and the recurrence rate is naturally higher in patients with a preserved hypophysial stalk. In addition, even among patients determined to have undergone total resection, the proportion of those without recurrence for 10 years ranged from 64% to 80%. While patients with recurrence require retreatment, the incidence of complications increases as the frequency of surgery increases. Thus, regarding the selection of surgical treatment for recurrent craniopharyngioma, mortality was significantly higher in patients who underwent a second or subsequent surgery than in those who underwent initial surgery. Even in patients who have undergone total resection, if craniopharyngioma recurs, the postoperative course will be poorer, and living a normal life will be difficult [8,16].

Radiotherapy has been used in patients in whom total resection cannot be achieved in the initial surgery and in those with recurrence [8,14-15]. However, no favorable long-term outcomes have been achieved by conventional radiotherapy or stereotactic radiosurgery with a gamma knife and other techniques because of the occurrence of adverse events and other reasons [9,17-19]. In this situation, hypofractionated radiotherapy was found to yield favorable treatment outcomes. However, frequent administration of the therapy, long treatment duration, and the possible occurrence of adverse events were the problems identified [20]. Since CyberKnife was introduced as a representative treatment device for stereotactic radiotherapy, extremely favorable outcomes and low incidence of adverse events have been reported [12]. Lee et al. reported that stereotactic radiotherapy demonstrated a recurrence prevention rate of approximately 90% and caused no complications [21]. 

Reports on the effects of the CyberKnife treatment on visual and pituitary functions have also increased in recent years. Puataweepong et al., who administered CyberKnife treatment to 100 consecutive patients with tumors proximal to the optic nerve, reported that the incidence of visual dysfunction was 0 [22]. Conti et al., who investigated damage to the hypothalamus, reported that the incidence of hypothalamic disorder after treatment was insignificant even after long-term observation [23]. Moreover, Yang et al. investigated prognosis with the extent of resection taken into account. They reported that in 442 patients who received additional CyberKnife treatment regardless of the extent of resection, neither progression-free nor overall survival at either 5 or 10 years was significantly different between patients who underwent gross total resection and those who underwent subtotal resection and received radiotherapy [24]. Furthermore, surgery preserving the hypothalamus was reported to clearly reduce postoperative obesity [25].

Based on these results, a recommended therapeutic strategy option for craniopharyngioma with respect to the quality of life of patients in the future may be a combination of resection to a limited extent to prevent surgical complications and immediate addition of CyberKnife treatment for residual tumor tissues, instead of insisting on aggressive surgical treatment aimed at total resection. We assumed that CyberKnife treatment should be considered before planning the resection of the recurrent tumors even if craniopharyngioma recurs in patients determined to have undergone total resection.

While we perform surgery using the extended transsphenoidal approach, we terminate resection when abrasion is difficult to perform under microscopic or endoscopic visualization, or when vascular injury or hypothalamic disorder is predicted because abrasion will be blindly performed even under endoscopic visualization. In principle, the pituitary gland and hypophysial stalk are always preserved. Under these policies, the total and subtotal resection rates are lower than those presented in various reports [26-27]. However, the ultimate outcomes are superior, and the incidence of complications is lower with CyberKnife.

The risks of hypopituitarism and visual dysfunction after CyberKnife treatment, which have been a concern, have been found to be insignificant in recent reports of long-term follow-up studies. Even in radiotherapy other than CyberKnife, these risks can be greatly avoided by fractionated irradiation. In the future, radiotherapy, which played an auxiliary role in the therapeutic strategy for craniopharyngioma, is expected to become a mainstream strategy. Among the patients included in this study, four were followed up for 10 years or more. We would like to continue follow-up of the patients and to examine the safety at 10 and 15 years.

## Conclusions

In patients who underwent safe surgery to a limited extent that did not cause any complications and received CyberKnife treatment for residual or recurrent tumors, the five-year long-term outcomes were extremely favorable. No complications due to CyberKnife treatment were observed after five years of follow-up. In the treatment of craniopharyngioma, a combination of palliative surgery and CyberKnife treatment may yield favorable outcomes in the future.
